# Applying the Bell’s Test to Chinese Texts

**DOI:** 10.3390/e22030275

**Published:** 2020-02-28

**Authors:** Igor A. Bessmertny, Xiaoxi Huang, Aleksei V. Platonov, Chuqiao Yu, Julia A. Koroleva

**Affiliations:** 1 School of Computer Science and Technology, Hangzhou Dianzi University, Hangzhou 310018, China; huangxx@hdu.edu.cn; 2 Saint Petersburg National Research, University of Information Technology Mechanics and Optics, St. Petersburg 197101, Russia; aplatonovv@gmail.com (A.V.P.); julietkoroleva@gmail.com (J.A.K.); 3 Beijing Institute of Technology, Beijing 100081, China; yuchuqiao@bit.edu.cn

**Keywords:** text mining, content analysis and indexing, text analysis

## Abstract

Search engines are able to find documents containing patterns from a query. This approach can be used for alphabetic languages such as English. However, Chinese is highly dependent on context. The significant problem of Chinese text processing is the missing blanks between words, so it is necessary to segment the text to words before any other action. Algorithms for Chinese text segmentation should consider context; that is, the word segmentation process depends on other ideograms. As the existing segmentation algorithms are imperfect, we have considered an approach to build the context from all possible n-grams surrounding the query words. This paper proposes a quantum-inspired approach to rank Chinese text documents by their relevancy to the query. Particularly, this approach uses Bell’s test, which measures the quantum entanglement of two words within the context. The contexts of words are built using the hyperspace analogue to language (HAL) algorithm. Experiments fulfilled in three domains demonstrated that the proposed approach provides acceptable results.

## 1. Introduction

The exponential growth in the number of Internet resources has led to problems in information retrieval. A simple search query can generate thousands or even millions of links. Traditional search methods are based on a selection from an index that links to documents that contain words from the query line [1]. The quality of a naive search using the same words from the query is insufficient for many reasons, including the synonymy and polysemy problems. For European languages, there are some techniques that improve the quality of information retrieval based on the index search. Latent semantic analysis (LSA) [2] allows finding words that have close meanings by using singular value decomposition. Hyperspace analogue to language (HAL) [3] analyzes word pairs by taking into account the frequency of each pair and the distance between words in the text. These models allow words to be represented by vectors of a reduced dimension, where semantically close words have similar vectors. Each row of the HAL matrix represents the context of a word that could be meant as a word sense. Information retrieval by word vectors instead of word patterns could involve word meanings into the search process and increase the search quality.

Searching by word vectors requires the documents to be vectorized. In this case, the information retrieval means that the document vectors should match the query vector. This matching is usually based on linear algebra. Nowadays, some researchers apply quantum mathematics to biology [4], sociology [5,6], and this task [7,8]. Document relevance to the query could be measured as a document vector’s projection to the query plane built by two query words. In [9,10], Bell’s test was used to assess the entanglement of words from a query in English texts as a measure of document relevance.

Chinese differs from alphabetic languages due to its very high context dependency because of the wide polysemy of ideograms. The coexistence of words in a text is not enough to assess their relevance to the query. Another feature of Chinese is the absence of blanks between words, which yields the nontrivial problem of word segmentation. The goal of this study is to estimate the applicability of quantum-inspired models to information retrieval in Chinese texts. This paper proposes an approach to estimate word pair entanglement using Bell’s test in CHSH form. In this work, an entanglement of two words means a correlation of these words’ contexts in documents. This measure is used for ranking Chinese documents by a pair of words from a user’s query.

## 2. Materials and Methods 

The wide polysemy of Chinese words makes searching documents by patterns inefficient. From this point of view, it is interesting to apply to Chinese texts an approach that was validated for English in [10]. This approach is based on vector word representation and it incorporates the context of words into the search process. The authors of [10] used quantum mathematical formalism and Bell’s test as a measure of documents’ relevance to the query. Unlike in [10], in this work, an asymmetric HAL built on the n-gram segmentation principle was used.

Vector document representation operates with words but not with symbols. Chinese words consist of one or more characters, but a text in Chinese is a continuous sequence of ideograms without spaces between words. The standard preprocessing stage of Chinese texts is word segmentation. There are several Chinese word segmenting programs. The simplest ones, such as JIEBA (https://pypi.org/project/jieba/), do not use a dictionary, but the segmentation quality is not high. More powerful Chinese text analyzers such as CRIE implement multiphase processing, including word segmentation based on a large dictionary [11]. The authors of [12] showed that Chinese word segmentation is able to improve the performance of information retrieval in Chinese texts, but the improvement is not significant if the search is based on words as patterns in the documents (i.e., substrings in the sequence of ideograms).

In [13], an approach to Chinese document retrieval based on the matching of text strings was considered. Relevance was determined by how terms appeared in the text collection rather than by statistics of appearance, and the order of words was taken into account. 

Unlike alphabetic languages, a separate syllable written as an ideogram in Chinese has a meaning. This allows the use of syllable-based statistical information in information retrieval [14]. Researchers have also had a positive experience in domain term extraction from Chinese texts without word segmentation [15]. The key idea is to split the sequence of symbols such as “abcdefg” into overlapping n-grams of “abc”, “bcd,” “cde,” “def,” and “efg.” Of course, among real words, this set will contain some bonded fragments of adjacent words. On the one hand, such pseudowords overload the document’s glossary; on the other hand, they indicate the joint occurrence of the words.

The “bag-of-words” paradigm limits the ability of a computer to understand natural language because it ignores the meaning of words and sentences. The vector model is an attempt to describe the sense of a word by the context [16]. Vector word representation means transforming a word w into the vector V_w_ = [v_1_ v_2_ … v_L_]^T^, which has dimension L in the space of real numbers. Thus, we need to build a projection a(w): W → R^L^, where W is a set of words, and w ∈W. According to the distributive hypothesis of Harris [7], v_i_ contains the number of occurrences of the word vi from the dictionary *W* in a window around the word w. So, for the sentence “Alice likes Bob” and the dictionary [Alice, like, Bob], the context of the word “like” is defined by the vector v = [1 0 1]^T^. Of course, the source text should be lemmatized before processing. This context representation indirectly, via the window size, takes into account the distance between words. A HAL matrix [3] allows reflection in one matrix cell as a frequency of pairs v_i_, v_j_ as the distance *d_p_* between these words in context *p* using the following formula:

(1) $$HAL_{i,j}=\overset{P_{ij}}{\underset{p=1}{\sum }}(S-d_{p}+1),\;d_{i,j}<S$$

where *S* is the size of the HAL window, and *P_i,j_* is the number of contexts of width *S* in which the words *v_i_*, *v_j_* are contained. Each context of width *S* means a sequence of words of length *S.* Thus, if two words are contained in the same context of width S, it means that they are contained in some word sequence and the distance between them does not exceed *S*. Each row of the HAL matrix is the word’s vector and the sum of all rows builds the document’s vector. 

The HAL matrix can be illustrated by a simple example. Let us consider the text “Alice likes Bob but Bob hates Alice.” The index after removal of the stop word “but” looks like this: [Alice:0, Likes:1, Bob:2, hates:3]. Assume the HAL window size is equal to 3. Then, the first row of the HAL matrix related to the word “Alice” is [0, 3, 2+1, 0], where 2+1 means two occurrences of pairs “Alice–Bob” with distances 2 and 3, respectively. Thus, the value in a cell of the HAL matrix reflects both the frequency of word pairs and the proximity of these words in the text. 

Matrix representation of words makes it convenient to apply the quantum probability theory that operates with density matrices [17]. Moreover, modern studies in sociology demonstrate that quantum probability theory is able to explain the violation of laws of classical logic when interviewing people [18]. In [19], an analogy was discovered between the well-known double-slit experiment with elementary particles and the user’s state in information retrieval. Natural language is highly contextual; that is, an information stream can be represented by the quantum framework where each word or phrase could be modeled by density operators [20].

The aim of our research was to range documents {D} according to their relevance to a query consisting of two words: *w*_1_ and *w*_2_. First, we built a word index for the domain. Without word segmentation, the word index means all n-grams presented in the domain documents. Second, we built a HAL matrix for each HAL window size. The HAL matrix had the dimensions *L* × *L*, where *L* is the dictionary length. The *j*th coordinate of the document vector was

(2) $$\psi_{j}=\overset{L}{\underset{i=1}{\sum }}AL_{i,j}.$$

Then, we used the formula for the Bell’s test [7]:

(3) $$S_{Bell}=\left|\langle \hat{A}{\hat{B}}_{+}\rangle {}_{\psi }+\langle {\hat{A}}_{x}{\hat{B}}_{+}\rangle {}_{\psi }\right|+\left|\langle \hat{A}{\hat{B}}_{-}\rangle {}_{\psi }-\langle {\hat{A}}_{x}{\hat{B}}_{-}\rangle {}_{\psi }\right|$$

where

(4) $${\hat{A}}_{x}=\left[\begin{array}{cc} 0 & 1\\ 1 & 0 \end{array}\right],\;{\hat{B}}_{x}={\hat{M}}^{-1}\cdot {\hat{A}}_{x}\cdot \hat{M},$$

(5) $$\;\hat{M}=\begin{array}{cc} p & -\sqrt{1-p^{2}}\\ -\sqrt{1-p^{2}} & p \end{array}$$

(6) $$\;{\hat{B}}_{+}=-\frac{\hat{B}+{\hat{B}}_{x}}{\sqrt{2}},\;{\hat{B}}_{-}=\frac{\hat{B}-{\hat{B}}_{x}}{\sqrt{2}},$$

(7) $$\hat{A}=\left[\begin{array}{cc} 1 & 0\\ 0 & -1 \end{array}\right],\;\hat{B}={\hat{M}}^{-1}\cdot \hat{A}\cdot \hat{M},$$

(8) $$\langle operato\rangle r_{\psi }=\langle bra\left|operator\right|ket\rangle ,$$

(9) $$bra=\left[\alpha \;\beta \right],\;ket=\left[\begin{array}{c} \alpha \\ \beta \end{array}\right]$$

where $=\langle u_{B}|u_{A}\rangle$, $u_{A,\;}\;u_{B}$ are the normalized vectors of query words *A* and *B*, and *α*, *β* are projections of the document vector to vectors $u_{A\;},\;u_{B}$. Each addend in formula (3) can obtain values in {–1,1}, so the maximal value of *S_bell_* = 4, but according to Tsirelson’s bound [20], it cannot exceed 2√2. Values between 2 and 2√2 mean a high correlation of the query words in the domain context.

**Figure 1:**
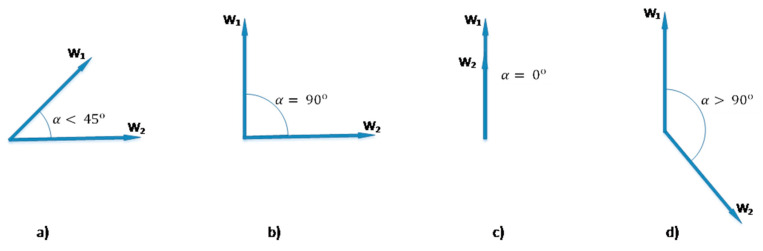
Graphic interpretation of Bell’s test.

A simple illustration of formula (3) is presented in the Figure 1, where $W_{1},{\;W}_{2}$ are the vectors of words. The case “a” can be considered as maximizing the expression (3). It shows the margin case for the formula $S_{\mathrm{bell}}=\left|\frac{\sqrt{2}}{2}+\frac{\sqrt{2}}{2}\right|+\left|\frac{\sqrt{2}}{2}-\left(-\frac{\sqrt{2}}{2}\right)\right|=2\sqrt{2}$. It violates Bell’s inequation and reflects a high semantic link of two words in the context. Cases “b” and “c” mean the negation and identity of words, respectively, and yield the value of the expression $S_{\mathrm{bell}}=2$. Any link missing between the words shows the “d” case and $S_{\mathrm{bell}}$ tends to be less than two.

## 3. Results

For the experimental data, we used the textbook “Geology“ (www.baike.com/wiki/地质学基础), where each chapter was considered as a separate document. Thus, these documents corresponded to the same domain but to different topics. The task of the experiment was to range these topics according to their relevance to the query consisting of two words: 火山 (volcano) and 岩石 (rock). Each query word consisted of two syllables, so we split the documents to overlapping 2-grams, as shown in Section 2. The statistics of query word occurrence are listed in Table 1.

**Figure 2:**
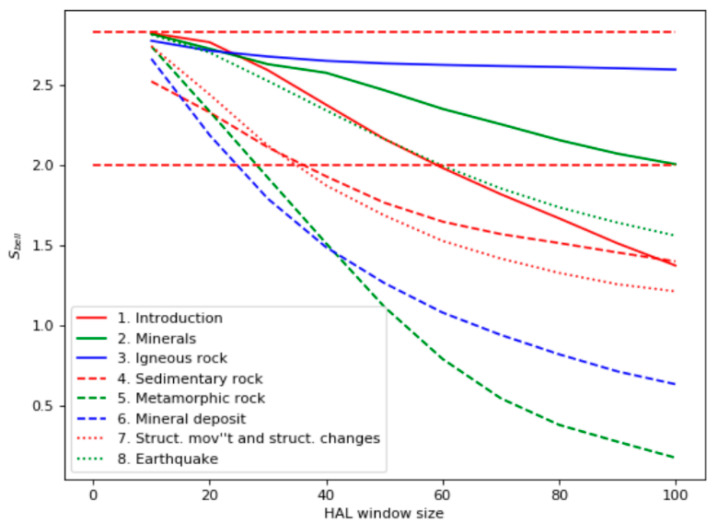
Bell’s test for the domain “Geology.”

According to the occurrence statistics, chapters 3, 6, and 7 should be the most relevant to the query. However, the Bell’s test curves shown in Figure 2 demonstrate different results: chapters 3, 2, 1, and 8 are the most relevant.

**Figure 3:**
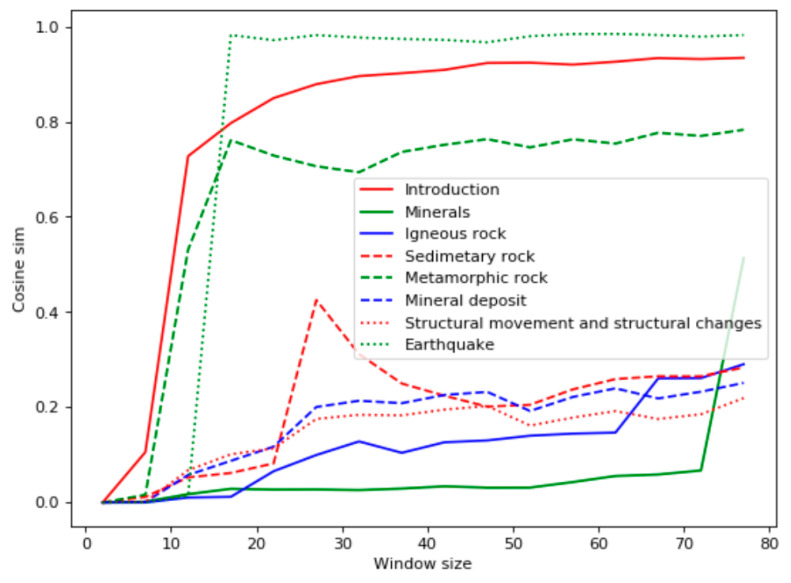
TF-IDF analysis for the domain “Geology.”

The most closely related to the query was the chapter “Igneous rocks,” which corresponded to the frequency of query words in the document. The chapter “Minerals” only had two occurrences of the word “volcano,” but it contained many facts about volcanic rocks. Ranked third and fourth were the chapters “Introduction” and “Earthquake,” which also had few instances of the query words but higher values of the Bell’s test. “Introduction” has many phrases related to different types of rocks, and “Earthquake” discusses volcanic activity, which is closely related to the topic “volcanic rocks.” On the contrary, the TF-IDF analysis (Figure 3), along with “Earthquake” and “Introduction,” highlighted as relevant the chapter “Metamorphic Rock,” which was not actually relevant. The TF-IDF measure is a numerical statistic that reflects how important a word is to a document in a corpus. This measure is often used as a weighting factor of words. It increases proportionally to the number of times a word appears in the document and decreases if the word is a general term (like conjunctions or stop-words). It this paper the TF-IDF statistic is used for context vectors with cosine distance for them as an alternative to the measure based on HAL and Bell’s test.

In the second experiment, we used the textbook “History of Natural Science” (book.douban.com/subject/3428495/), which was also divided into eight topics. The occurrences of the query words 实验 (experiment) and 科学 (science) are shown in Table 2. Based on the query word frequency, documents 7, 5, and 4 were expected to be the most relevant.

**Figure 4:**
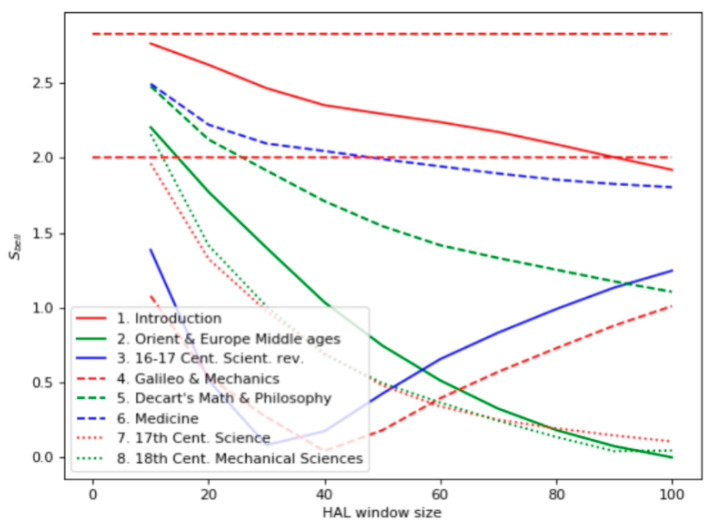
Bell’s test for the domain “History of Science.”

Like the first experiment, the Bell’s test results (Figure 4) differed from the ones based on the term frequency: the most relevant documents were found to be 1, 6, and 5. Similarly, the chapter “Introduction” contains many sentences related to scientific methods, including experiments. The high level of the Bell’s test for the chapter “Medicine” reflected the empirical nature of early medical research. The chapter “Descartes’s Math and Philosophy” appeared irrelevant to the query, even though it contains a description of some philosophical mental experiments. Chapters 2 and 3 have many facts about experiments, but they are not explicitly indicated as scientific ones.

**Figure 5:**
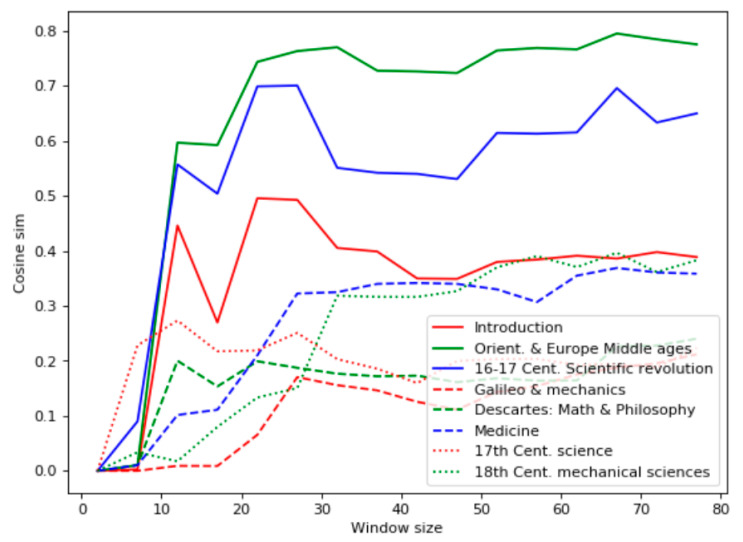
TF-IDF analysis for the domain “History of Science.”

The TF-IDF analysis of the domain “History of Science” demonstrated significant nonmonotonic behavior, as shown in Figure 5. These data cohered with the term occurrences listed in Table 2 but did not reflect the sense of the chapters. Particularly, according to the TF-IDF analysis, the most relevant chapter is “Orient. and Europe Middle Ages,” which contains only a few references to scientific experiments. 

**Figure 6:**
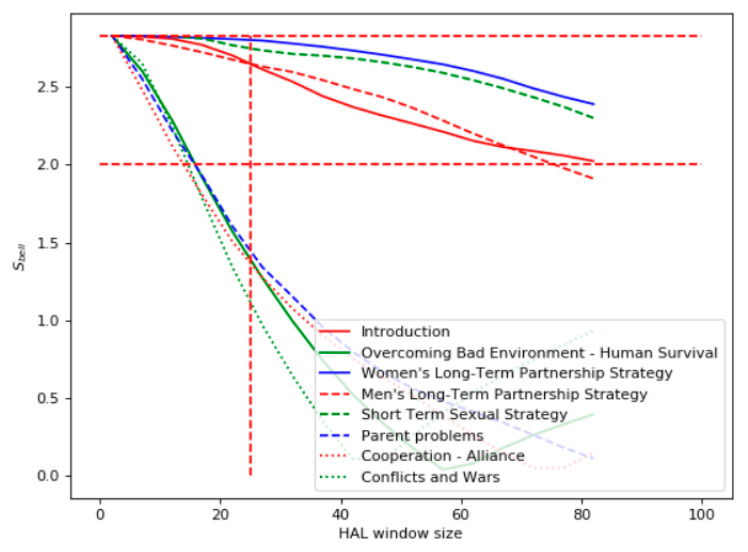
Bell’s test for the domain “Psychology.”

The third domain considered by the experiments was “Psychology” and the source of the texts was the Chinese version of the book “Evolutionary Psychology” by David Buss [21]. Figure 6 demonstrates the results of the Bell’s test for the query “natural selection.” One can see that the highest violation of the Bell’s test was for the book chapters “Women’s Long-Term Partnership Strategy,” "Short-Term Sexual Strategy," "Men’s Long-Term Partnership Strategy," and “Introduction.” The chapters "Conflicts and Wars," "Parent Problems," "Overcoming Bad Environment—Human Survival," and "Cooperation–Alliance" had the lowest relevance. This ranking was satisfactory according to the expert assessment.

**Figure 7:**
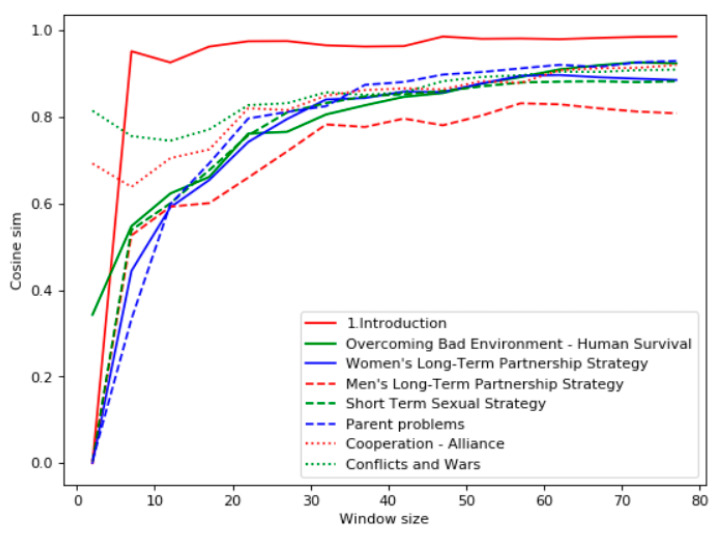
TF- IDF analysis for the domain “Psychology.”

According to the TF-IDF analysis within the window (Figure 7), the most relevant chapter to the query “natural selection” is “Introduction.” Other chapters had quite close TF-IDF values. The minimal TF-IDF value was for the chapter “Men’s Long-Term Partnership Strategy.”

The comparison of the Bell’s test and TF-IDF analysis allowed us to conclude that Bell’s test distinguishes relevant and nonrelevant topics more clearly and properly than TF-IDF. The table below shows TF-IDF and Bell’s test comparison to summarize this observation. Table 3 contains the computed discounted cumulative gain (DCG) measure for each domain of the experiment. This measure shows the ranking quality of an algorithm. The formula for the DCG measure is

$$\;DCG=\overset{p}{\underset{i=1}{\sum }}\frac{rel_{i}}{log_{2}\left(i+1\right)}$$

where *p* is the number of documents (or positions), *i* is position of a document in order of algorithm estimation, and *rel_i_* is the estimation of relevance for the *i*th position (it can be 0 for an irrelevant document or 1 for a relevant document).

In accordance with our experiment, Bell’s test as an algorithm for relevance estimation showed a greater value of the DCG measure than the TF-IDF algorithm. This experiment demonstrates the validity of using Bell’s test for document ranking. A comparison of Bell’s test as a ranking algorithm and other algorithms (not only TF-IDF) with bigger test samples can be considered as a direction for further research.

## 4. Discussion

The Bell’s test applied to a pair of words in a document is a measure of the “entanglement” of two words in the text. The results of our experiments for Chinese texts demonstrate that the Bell’s test, in general, allows ranking the documents according to their relevance to the query. Unlike the results found for English texts [6], where the values of Bell’s test increased by expanding the HAL window, the Bell’s test for Chinese texts demonstrated the opposite behavior. In a narrow context, all the documents had the value S_bell_ > 2. This phenomenon could be explained by the wide polysemy of ideograms where narrow contexts (short HAL window) look similar. This effect is strengthened because of the avoidance of word segmentation where fragments of words are considered as separate words. With a wider context, the values of Bell’s test for the considered documents differed. Thus, the criterion of the relevance of a document to a query could be the point of crossing the Bell’s curve at the line *S_bell_* = 2. The later this point, the more relevant the document.

## 5. Conclusions and Future Work

The experimental study on two subject areas demonstrated the validity of Bell’s test for Chinese texts without word segmentation with one restriction: Bell’s test is not valid for a narrow context. The Bell’s test is informative for HAL window size values greater than 20-30. The advantage of Bell’s test for Chinese texts is its ability to find relevance for a query even for documents containing a few instances of the query words. The disadvantage of the proposed approach is the necessity of using, as a query, only a couple of n-grams of the same length.

Future work might improve the method of calculation of the document vector. Presently, it is implemented as the sum of rows of the HAL matrix (2). Further research should also emphasize searches using more than two-word queries. The current approach is based on the quantum-like entanglement of two query words, similar to how quantum mechanics deals only with two particles. Another problem that is not discussed here is the search speed [20]. The time it takes to build the document’s vector is several seconds and this is critical if one were to process many documents.

## Figures and Tables

**Table 1:** Term occurrence in the documents of the domain “Geology.”

No.	Document	火山 (volcano)	岩石 (rocks)
1	Introduction	7	51
2	Minerals	2	26
3	Igneous rock	238	122
4	Sedimetary rock	38	193
5	Metamorphic rock	4	148
6	Mineral deposit	44	48
7	Structural movement and structural changes	43	44
8	Earthquake	18	31

**Table 2:** Term occurrence in the documents of the domain “History of science.”

No	Document	实验 (experiment)	科学(science)
1	Introduction	6	51
2	Orient. and Europe Middle ages	19	73
3	16th and 17th Cent. Scientific revolution	19	46
4	Galileo and mechanics	30	27
5	Descartes’s math and philosophy	35	110
6	Medicine	19	56
7	17th Cent. science	58	189
8	18th Cent. mechanical sciences	6	44

**Table 3:** Computed discounted cumulative gain for each domain.

Domain Name	TF-IDF DCG	Bell’s Test DCG
Geology	1.08	1.70
History of science	1.24	2.13
Psychology	1.67	2.38

## References

[1] Dong H., Hussain F., Chang E. A survey in traditional information retrieval models. Proceedings of the 2008 2nd IEEE International Conference on Digital Ecosystems and Technologies.

[2] Dumais S.T. (2005). Latent Semantic Analysis. Ann. Rev. Inf. Sci. Technol..

[3] Jones M.N., Willits J., Dennis S. (2015). Models of Semantic Memory. The Oxford Handbook of Computational and Mathematical Psychology.

[4] Arndt M., Juffmann T., Vedral V. (2009). Quantum Physics Meets Biology. HFSP J..

[5] Arfi B. (2018). Challenges to a Quantum-Theoretic Social Theory. Millenn. J. Int. Studies.

[6] Haven E., Khrennikov A. (2013). Quantum Social Science.

[7] Busemeyer J.R., Bruza P.D. (2012). Quantum Models of Cognition and Decision.

[8] Khrennikov A., Aerts D., Khrennikov A., Melucci M., Toni B. (2019). Basics of Quantum Theory for Quantum-Like Modeling Information Retrieval.

[9] Barros J., Toffano Z., Meguebli Y., Doan B.L., Atmanspacher H., Haven E., Kitto K., Raine D. (2014). Contextual Query Using Bell Tests.

[10] Platonov A.V., Bessmertny I.A., Semenenko E.K., Alodjants A.P., Aerts D., Khrennikov A., Melucci M., Toni B. (2019). Non-Separability Effects in Cognitive Semantic Retrieving.

[11] Sung Y.T., Chang T.H., Lin W.C. (2016). CRIE: An automated analyzer for Chinese texts. Behav. Res..

[12] Zhihan L., Xu Y., Geva S. A Hybrid Chinese Information Retrieval Model. Proceedings of the 6th International Conference on Active Media Technology.

[13] Zeng B., Yao L., Wang R. A Chinese Document Retrieval Method Considering Text Order Information. Proceedings of the 2017 International Conference on Computer, Electronics and Communication Engineering.

[14] Bai B.R., Chen B., Wang H.M. (2000). Syllable-based chinese text/spoken document retrieval using text/speech queries. Int. J. Pattern Recognit. Artif. Intell..

[15] Yu C., Bessmertny I.A. Contrastive Domain Term Extraction from Chinese Texts without Word Segmentation. Proceedings of the International Conference on Advanced Education and Management Sciences (AEMS-2017).

[16] Turney P.D., Pantel P. (2010). From Frequency to Meaning: Vector Space Models of Semantics. J. Artif. Intell. Res..

[17] Wittek P., Darányi S., Song D., Melucci M., Frommholz I., Zhang P., Wang L., Arafat S. (2011). Spectral Composition of Semantic Spaces.

[18] Meguebli Y., Popineau F., Doan B.L. A Novel Architecture for a Smart Information Retrieval System Based on Opinions Engineering. Proceedings of the Symposium on Human-Computer Interaction and Information Retrieval.

[19] Zuccon G. An analogy between the double slit experiment and document ranking. Proceedings of The 3rd IRSG Symposium: Future Directions in Information Access.

[20] Melucci M. (2019). An Efficient Algorithm to Compute a Quantum Probability Space. IEEE Trans. Knowl. Data Eng..

[21] David B. (2011). Evolutionary Psychology: The New Science of the Mind.

